# Nanoscale shape-dependent histone modifications

**DOI:** 10.1093/pnasnexus/pgac172

**Published:** 2022-08-27

**Authors:** Wei Zhang, Jingji Li, Camila P Silveira, Qi Cai, Kenneth A Dawson, Gerard Cagney, Yan Yan

**Affiliations:** Guangdong Provincial Education Department Key Laboratory of Nano-Immunoregulation Tumor Microenvironment, The Second Affiliated Hospital, Guangzhou Medical University, Guangzhou 510260, Guangdong, P.R. China; Centre for BioNano Interactions, School of Chemistry, University College Dublin, Belfield, Dublin 4, Ireland; Centre for BioNano Interactions, School of Chemistry, University College Dublin, Belfield, Dublin 4, Ireland; Centre for BioNano Interactions, School of Chemistry, University College Dublin, Belfield, Dublin 4, Ireland; Centre for BioNano Interactions, School of Chemistry, University College Dublin, Belfield, Dublin 4, Ireland; Guangdong Provincial Education Department Key Laboratory of Nano-Immunoregulation Tumor Microenvironment, The Second Affiliated Hospital, Guangzhou Medical University, Guangzhou 510260, Guangdong, P.R. China; Centre for BioNano Interactions, School of Chemistry, University College Dublin, Belfield, Dublin 4, Ireland; School of Biomolecular and Biomedical Science, UCD Conway Institute of Biomolecular and Biomedical Research, University College Dublin, Belfield, Dublin 4, Ireland; Centre for BioNano Interactions, School of Chemistry, University College Dublin, Belfield, Dublin 4, Ireland; School of Biomolecular and Biomedical Science, UCD Conway Institute of Biomolecular and Biomedical Research, University College Dublin, Belfield, Dublin 4, Ireland

**Keywords:** nanoscale shape, histone modification, gold nanoparticle, epigenetics, bionanosynapse

## Abstract

Recent observations suggest a role for complex nanoscale particulate shape in the regulation of specific immune-related cellular and in vivo processes. We suspect that cellular recognition of nanostructure architecture could involve nonmolecular inputs, including cellular transduction of nanoscale spatially resolved stresses induced by complex shape. Here, we report nanoscale shape-dependent control of the cellular epigenome. Interpretation of ChIP-Seq sequencing suggests that differential marking of H3K27me3 may be linked to sensory and synapse-recognition of nanoscale forces induced by complex shape. The observations raise significant questions on the role of particle-shape-induced immune regulation and memory, with potential consequences in both causes and treatment of immune-related disease.

Significance statement While mechanisms remain sketchy, a long legacy of epidemiological and biological investigation suggests the immune system can capture and memorise extended integrated information about (endogenous and exogenous) nanoscale structures. Those ideas have been sharpened by recent observations that nanoscale pathogens or particulates lead to nonspecific (antigen-independent) innate immune memory. Here, we identified a range of changes in histone modifications in response to nanoscale shape using proteomics. Interpretation of H3K27me3 ChIP-Seq, one of the shape-dependent differential histone modifications, suggests that these epigenetic changes are linked to sensory and synapse-recognition of nanoscale forces induced by complex shape.

Several years ago we observed that (within certain regimes) complex nanoscale shape variations on the size scales of several nanometres could be reliably and reproducibly linked to interesting immune-related cellular and in vivo readouts (1, 2). The reader may consult the original articles for details, but we briefly summarise the background here (1–3). Statistically, highly reproducible ensembles of particles made using flow chemistries are studied by image capture of electron micrographs (1–3). Computational analysis (analogous to Fourier decomposition) of the particle contours is followed by principal component analysis to rank prominent contributions (in descending order, PC1, PC2, etc.) that describe the differences in the different shape ensembles. While this description is linked to obvious structural properties (such as the numbers of “spikes” on the particle and the inter-spike distances), it embodies much more structural information. The approach allows a firm reproducible linkage between structure and biological function, and suggests that specific quite narrowly defined “hot spot” shape ensemble regimes could regulate immune-related responses independently of (or in combination with) specific biomolecular surface recognition. In this study, GNP2 is representative of the particle “spiky” regime that exhibits the interesting immune-related effects previously reported. Shapes in such regimes are expected to induce large spatially resolved stress patterns across particle-cell membrane interfacial domains of several tens of nanometres. Analytical studies reveal no chemical differences in the bare particle surfaces of different shapes, and biomolecular surface coatings are maintained free from pyrogens or other known adjuvants. Still, after repeated in vivo doses, we observe shape-differentiated effects (including antibodies to self-antigens) in the absence of other known biomolecular stimuli. While we are investigating a number of potential underlying mechanisms, including shape-induced transduction of nanoscale stresses, there is as yet no broad and conclusive mechanistic understanding. Nevertheless, as part of these studies, we observed (and have now investigated in some detail) the particle shape dependence of cellular epigenomes. In light of our previous findings, we believe the outcomes are sufficiently intriguing to be reported and illustrated here.

In brief, gold nanoparticles were synthesized in a clean environment, found to be liposaccharide (LPS) free, and characterized by a range of techniques to determine their physiochemical properties. Two distinct representative shapes, GNP1 (a conventional spherical like nanoparticle) and GNP2 (drawn from the shape regime of interest identified in our recent studies), are discussed here. These shapes are characterized using our previously reported image-computational analysis approach to shape capture based on transmission electron microscopy (TEM) images (Fig. 1a and b). This means of capturing shape detail is supplemented by conventional UV–Vis absorbance spectra (Fig. 1c) and differential centrifugation sedimentation, which while being shape-dependent, merely fingerprint without explicit capture of nanoparticle shape. Particles are found to have similar nominal zeta potentials (see the Supplementary Material), though the meaning of the Stern layer is hard to interpret for complex shapes. As usual in such studies, particles are dispersed in 100% human serum to form a hard surface corona and subsequently re-dispersed in cell culture media. The gold nanoparticles used in this study are immunologically clean, colloidally stable in relevant biological media (Fig. 1d), and we worked in conditions where cell death and overt cell damage were essentially undetectable. Cellular uptake studies were carried out on a human macrophage model (dTHP-1, obtained by differentiation of a human leukemia monocytic cell line) and human lung adenocarcinoma epithelial cell line (A549). In contrast to well-known nanoparticle accumulation patterns for GNP1, GNP2 nanoparticles showed unusual intracellular patterns in which they appeared to follow the contours of internalized membranes, and the interior of vesicles (including lamellar bodies for A549 cells) (Fig. 1e).

**Fig. 1.**
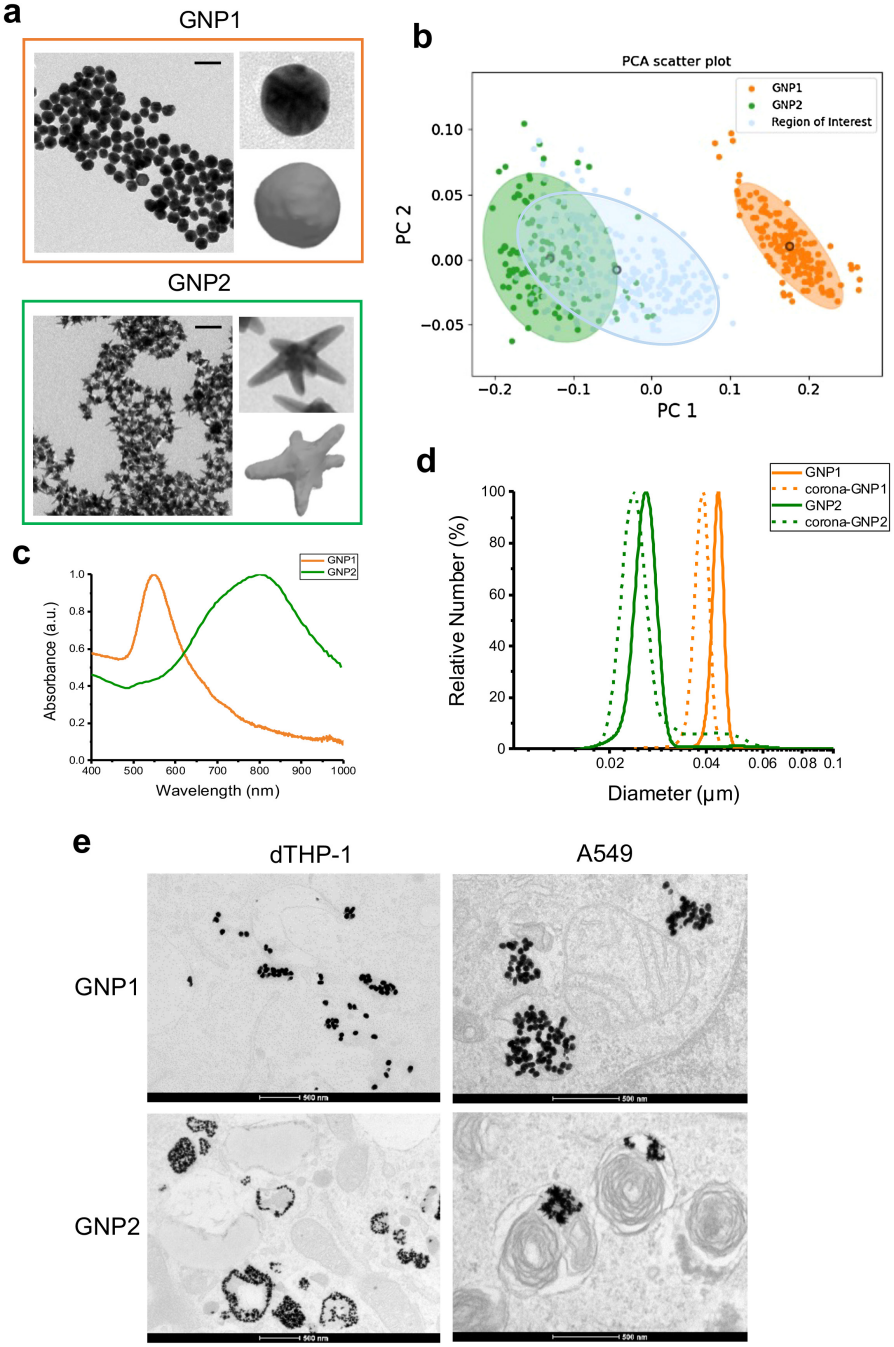
Biophysical characterization of gold nanoparticles with two distinct nanoscale shapes. (a) TEM micrographs of GNPs, scale bar 100 nm. Enlarged TEM images and shape contour 3D models are shown. (b) 2D scatter plot of GNPs from the shape contour analysis. GNP2 overlaps with the shape region of interest identified previously. (c) UV–Vis absorption spectra analysis. (d) Differential centrifugal sedimentation (DCS) analysis of GNPs and corona-GNPs. (e) TEM images of GNP1 or GNP2 internalized by dTHP-1 and A549 cells.

Consistent with our previous report, the transcriptome of dTHP-1 cells showed shape-dependent global responses, including overrepresented Gene Ontology (GO) terms related to histone modifications, after incubation with GNP1 or GNP2 for 24 h (Fig. 2a and b). We therefore employed a bottom-up mass spectrometry (MS) based strategy to comprehensively profile methylation and acetylation motifs on histones. After nanoparticle treatment histones (H2A, H2B, H3, and H4) were extracted and purified, derivatized with deuterated acetic anhydride, trypsinized, and analyzed by liquid chromatography (LC)-MS (4). The relative abundances of the modified peptides were compared to the untreated cells (as the control group) and expressed in a heatmap (Fig. 3c). All the significant changes (two-tailed *t*-test) detected in methylation and acetylation induced by GNPs are harbored in histone H3, and GNP1 and GNP2 are shown to differentially affect H3 methylation and acetylation. GNP2-induced demethylation of di- and tri-methylation lysine 27 (H3K27me2/3) (see the MS2 spectrum of H3K27me3 and the extracted ion signal in Fig. 2d and e). The decrease in H3K27me3 was validated by ELISA (Fig. 2f) and western blot (Fig. 2g). The same significant GNP2-induced demethylation of H3K27me3 was observed in other nondifferentiable (including A549) cell lines (Fig. 2h). We then investigated the epigenomic effect further using chromatin immunoprecipitation sequencing (ChIP-seq).

**Fig. 2.**
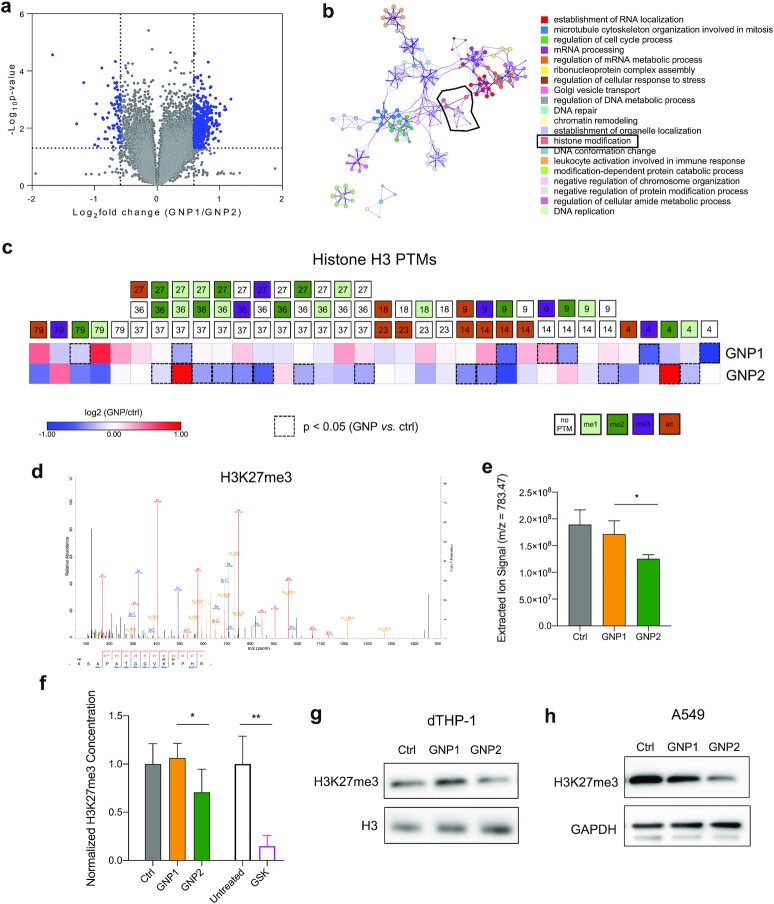
Nanoscale shape-dependent effects on histone modifications. (a) Volcano plots of transcriptomics to show differentially expressed genes (DEGs, in blue dots) between GNP1 and GNP2 treated cells. (b) GO term enrichment analysis (biological processes) and cluster analysis (Metascape) of the DEGs, highlighting histone modification. (c) Heatmap showing hierarchical clustering analysis of GNP1- and GNP2-induced methylation and acetylation on histone H3. (d) MS/MS spectrum of −27KSAPATGGVKKPHR40- peptide with tri-methylation on Lys27 (H3K27me3) and (e) the extracted ion signals of H3K27me3 peptides. *P <  0.05 (*t*-test). (f) Concentration of H3K27me3 quantified by ELISA. Data of untreated or ctrl were set as 1. GSK treated cell lysate was used as a positive control. *P <  0.05; **P <  0.01 (*t*-test). Western blot of H3K27me3 from dTHP-1 (g) and A549 (h) cells after the treatment with GNP1 or GNP2 for 24 h.

**Fig. 3.**
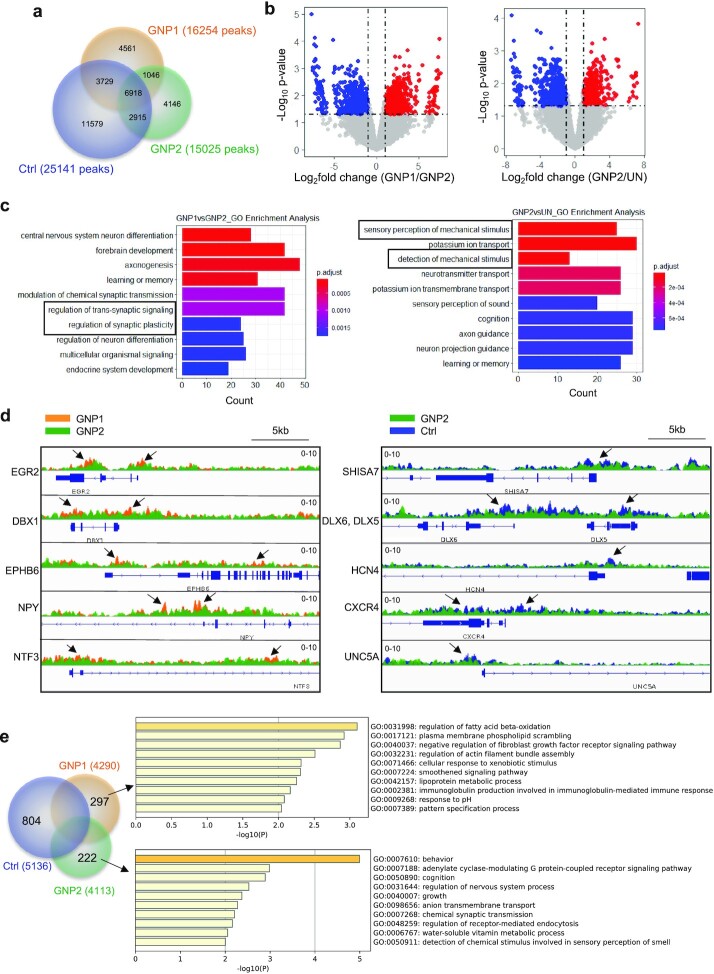
Genome-wide differential distribution of H3K27me3 maps to pathways associated with sensory and synapse function. (a) Venn diagram showing the number of consensus peaks of H3K27me3 in ChIP-seq data sets. (b) Volcano plot of the differential H3K27me3 sites. (c) GO enrichment analysis of the differential H3K27me3 sites after annotation with genes. (d) Examples of gene feature tracks showing the differential H3K27me3 peaks, and the associated genes. (e) Venn diagram showing the number of genes identified from the H3K27me3 consensus peaks for each treatment group. The GO term enrichment analysis of the uniquely marked genes for GNP1 and GNP2.

ChIP-qPCR with known H3K27me3 negative and positive primers was used to determine the optimal antibody concentration to improve signal to background ratio. For ChIP-Seq sequencing, we chose 50 million reads for each sample as H3K27me3 involves large enrichment domains and employed MACS3 (5) for peak calling. We identified, respectively, 25,141 (control), 16,254 (GNP1), and 15,025 (GNP2) consensus peaks from two biological repeats (Fig. 3a). Direct analysis of peaks suggested significant differential effects between the particles and control, and between the two types of particles (Fig. 3b). Simple GO-term enrichment analysis suggested the differential H3K27me3 peaks for GNP2 (in comparison to GNP1 or untreated) are strongly associated with trans-synapse signalling and mechanical stimuli (Fig. 3c). Differential H3K27me3 peaks (see the arrows in Fig. 3d) that are annotated with sensory and synapse-related GO terms are exemplified in Fig. 3d. In addition, a “gene centric” approach was used to annotate genes of consensus peaks across the treatment groups, and, respectively, 5136 (control), 4290 (GNP1), and 4113 (GNP2) genes were annotated (Fig. 3e). We first observed significant differences between the untreated control and the different shapes. For instance, respectively 804 (control), 297(GNP1), and 222 (GNP2) genes had unique H3K27me3 marks that were not shared by the other treatment groups. For GNP1, these were mainly associated with GO terms linked to metabolism, whereas for GNP2, they are again related to “sensory and synapse” processes. Evidently, these ontological associations, while originally codified in relation to neuronal mechanisms, in the present context refer to analogous correlations in biomolecular species, events, and processes present in different cellular phenotypes. The implied linkage between (nanoscale-shape-induced) cellular forces, “synaptic recoding,” and synaptic transmembrane signalling is particularly intriguing because it is consistent with our developing understanding of the mechanisms of recognition and processing of such nanostructures via extended “information-rich” synapses formed at the interface between nanostructures and cellular membranes (6).

In summary, while numerous biomolecular species are reported to impact the epigenome, here we observe that epigenomic control may be exerted by some classes of nanoparticle shape for nominally benign materials and endogenously derived biomolecular coatings. While mechanisms or firm causal linkage remain elusive, a long legacy of informed scientific and epidemiological thought contemplates a link between endogenous biological debris or exogenous complex particulate pollutants and immune dysregulation, allergic or autoimmune diseases (7–11). We also draw attention to observations throughout the literature that externally applied microscopic cellular forces induce immunological control and the observation that nanoscale pathogens or particulates can lead to nonspecific (antigen-free) prolongation of innate immune memory (12–15). Our present report shows that distinct nanoscale architectures can reprogram the cellular epigenome, and suggests the underlying connection with “synaptic” recognition of spatially resolved cellular stresses. This could represent a scientific nexus linking all these ideas together. Certainly, based only on the present results, it would be premature to draw firm conclusions beyond the facts reported, but they do point toward a possible link between particle architecture and epigenomic immune memory. The potential to locally reprogram epigenomes, in close association with defined antigens, has significant implications ranging from adjuvant design in vaccine development to immunotherapies for reprogramming autoimmune and other diseases.

## Supplementary Material

pgac172_Supplemental_File

## Data Availability

Microarray transcriptome, proteomics for histone modification, and H3K27me3 ChIP-seq datasets generated during the current study are available in the Open Access Zenodo Repository, DOI: 10.5281/zenodo.7015170.
