# Supplementary material for: Nanoscale shape-dependent histone modifications
Source: PNAS Nexus. 2022 Aug 27;1(4):pgac172. doi: 10.1093/pnasnexus/pgac172 (PMC9802115; doi:10.1093/pnasnexus/pgac172)
Supplement: pgac172_Supplemental_File [file pgac172_supplemental_file.docx]

**
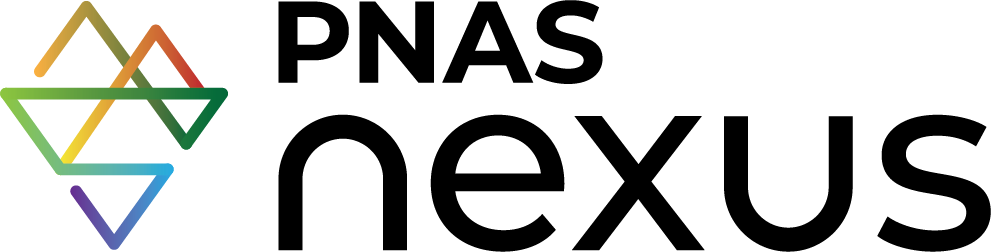
**

**Supplementary Information for**

Nanoscale Shape-Dependent Histone Modifications

Wei Zhang^a,b,§^, Jingji Li^b,§^, Camila P. Silveira^b^, Qi Cai^b^, Kenneth A. Dawson^a,b^, Gerard Cagney^c,^* & Yan Yan^b,c,^*

a. Guangdong Provincial Education Department Key Laboratory of Nano-Immunoregulation Tumor Microenvironment, The Second Affiliated Hospital, Guangzhou Medical University, Guangzhou 510260 Guangdong, P.R. China

b. Centre for BioNano Interactions, School of Chemistry, University College Dublin, Belfield, Dublin 4, Ireland

c. School of Biomolecular and Biomedical Science, UCD Conway Institute of Biomolecular and Biomedical Research, University College Dublin, Belfield, Dublin 4, Ireland

*Gerard Cagney, Yan Yan

^§^These authors contributed equally.

**Email:**  [gerard.cagney@ucd.ie](mailto:gerard.cagney@ucd.ie); [yan.yan@cbni.ucd.ie](mailto:yan.yan@cbni.ucd.ie)

**This PDF file includes:**

Supplementary Text

SI References

**Supplementary Information Text**

**NPs Synthesis.** Two shapes of gold nanoparticles (GNPs) were prepared following the methods described in literature (1). After synthesis, ligand exchange reaction was processed on GNP1 and GNP2 with Bis(*p*-sulfonatophenyl)phenylphosphine dehydrate dipotassium salt (BSPP).

**NP Characterization.** Characterisation of the gold nanoparticles were performed by Transmission electron microscopy (TEM), Nanoparticle Tracking Analysis (NTA), Ultraviolet-visible spectroscopy (UV-Vis), Dynamic Light Scattering (DLS) and Differential Centrifugal sedimentation (DCS). The shape and size of the nanoparticles were confirmed by TEM images. Computational identification of shapes was performed following a published procedure (2). Surface area was calculated based on the extracted contour during the computational shape analysis. The concentration of gold nanoparticles was defined as number of particles in certain volume (particles/ml), which was determined by NTA. Distribution and stability of gold nanoparticles in biological medium was checked by DLS and DCS analysis. The zeta potential of GNP1 and GNP2 in 1 × PBS buffer (pH = 7.4) was measured to be -38 ± 1 mV and -34 ± 2 mV, respectively.

**Endotoxin Contamination Control** Endotoxin level in all materials involved in cell culture was examined by Pierce LAL Chromogenic Endotoxin Quantitation Kit (Thermo Fisher). 250 μl of cell culture were centrifuged at 300 rcf and the supernatant were collected for testing. Gold nanoparticles were diluted to 1×10^11^ particles/ml with endotoxin-free water prior to testing. The testing was performed according to manufacturer’s instruction.

**Cell Culture** THP-1 cell line (DSMZ) were maintained at 1×10^5^ to 8×10^5^ cells/ml in RPMI 1640 medium (Gibco) supplied with 10% heated-inactivated (56 ℃, 30 min) Fetal Bovine Serum (FBS, Gibco), 100 units/ml of penicillin and 100 μg/ml of streptomycin (Gibco) in 5% CO_2_ at 37 ℃. THP-1 cells (4 × 10^5^ cells/ml) were differentiated using 100 nM PMA for 48 h. The PMA-treated cells were washed with fresh growth medium and incubated in growth medium for 24 h prior to nanoparticle treatment. GNPs were pre-coated by incubating with 100% heated-inactivated human serum (Sigma-Aldrich) for 1 hour at 37 ℃. Pre-coated GNPs were re-dispersed in macrophage serum-free medium (Gibco) to working concentration before applied to the cells. A549 cell line (ATCC CCL-185) were cultured in MEM (Life Technologies) supplied with 10% FBS, 50 units/ml of penicillin and 50 μg/ml of streptomycin (referred to hereafter as cMEM) in 5% CO_2_ at 37 ℃.

**TEM** Cells were seeded in 24-well plate with glass coverslip at the bottom. Nanoparticle-treated cells were washed with PBS and fixed with 2.5% glutaraldehyde in 0.1 M Sorensen’s phosphate buffer at 4 ℃ and preliminarily stained with 0.1% OsO_4_ for 1 hour. Dehydrating of cells were processed by exposing to 30%, 50%, 70%, 90% and 100% (twice) ethanol for 10 min each. The glass coverslip holding cells were dip to propylene oxide and covered with TAAB Epon resin 812, which was prepared by mixing 12 g Agar 100 resin, 4.75 g Dodecenyl Succinic Anhydride (DDSA), 8.25 g Methyl Nadic Anhydride (MNA) and 0.5 g 2,4,6-tri (dimethylaminoethyl) phenol (DMP-30) and incubated at 37 ℃ for 2 hour to allow Epon infiltration. The Epon was polymerised by baking at 60 ℃ overnight. 80 nm sections were produced by Ultramicrotome (Leica EM UC6) and diamond knife (DiATOME). The sections were post-stained with 2% uranyl acetate for 20 min and lead citrate for 2 min before viewing under FEI Tecnai 120.

**Transcriptome Microarray** THP-1 monocytes were seeded at 2.5×10^5^ cells/ml in 6-well plate and differentiated into THP-1 macrophages as described above, before nanoparticles exposure. Heated-inactivated human serum coated gold nanoparticles were dispersed in medium at concentration of 1 × 10^11^ particles/ml before applying to cells. After 24-hour treatments, the cells were washed with ice-cold PBS for twice. RNA of each well was extracted by Invitrap® Spin cell RNA mini kit following manufacturer’s instruction. The concentration and purity of RNA was examined by Nanodrop. RNA integrity number (RIN), which indicates the quality of RNA was determined by Bioanalyser (Aligent 2100) on Aligent RNA 6000 Nano kit. 20 μl of RNA was transferred into the center of each RNA stable tube (Merck), which is designed for long-term preservation of RNA samples at room temperature. The samples were then dried using a vacuum concentrator without heat for around 30 min and stored in a heat-sealed pouch with a desiccant packet provided with the tubes. Transcriptome microarray was performed by Inge Nelissen (VITO NV, Belgium).

**Proteomics** Histone post-translational modification analysis was carried out with a quantitative proteomic strategy described by Feller, et.al. in 2015 (3). Nanoparticle treated cells were collected by cell scrapper after washing with ice-cold PBS. Crude extraction of histones with other acid-soluble proteins were processed by suspending cell pellets in 0.2 M sulphuric acid overnight. Histone extracts were precipitated with 26% TCA and re-suspended in neutralised buffer (pH 8.0) after 3 times washes with ice-cold acetone. Further histone purification was carried out by separating crude extracts on 15% SDS-PAGE gel. The gels were stained with Coomassie blue and bands between 22 kDa and 11 kDa were isolated from the gels. Gel pieces were washed twice with HPLC water and twice with 100 mM Ammonium bicarbonate buffer (ABC buffer). Distaining of the gel pieces were processed by washing three times with 50 mM ABC/50% ACN buffer for 10 min with shaking at 37 ℃. The decoloured gels were incubated with 100 mM and 20 mM ABC for 15 min respectively and incubated three times with 100% ACN for 10 min to be dehydrated. 10 μl of D3AA were added to dehydrated gels followed by another 20 μl of 100 mM ABC buffer. Another 70 μl of 100 mM ABC were added to cover the gel pieces and incubated for 45 min at 37 ℃ for chemical acetylation. The pH of the reaction was adjusted to pH 7.0-8.0 with 1 M ABC buffer at 5 min after the start of incubation. After acetylation, the gel pieces were washed and incubated with 100 Mm ABC buffer and ACN for dehydration. Digestion was carried out by incubating the dehydrated gel pieces with Trypsin overnight at 37 ℃. Peptides were recovered by incubating two times with 70% ACN/0.25% TFA for 10 min at 37 ℃ followed by two times with 100% ACN. The harvested peptides were concentrated and harvested I 0.1% TFA. The purification and desalting of the peptides were carried out with 100 µL bed Pierce™ C18 tips (Thermo Fisher Scientific). The samples were injected into the Dionex Ultimate 3000 UPLC (Thermo Fisher scientific) connected to a Thermo Scientific Q-Exactive mass spectrometer. LC-MS data was analysed by Maxquant software (version 1.5.3.3) and Xcalibur software (Thermo Fisher Scientific).

RAW mass spectrometry files were analyzed using MaxQuant (version number 1.5.8.3). A Uniprot FASTA amino acid sequence database (release 2016_06; 70,317 entries) was used for MS/MS searches. False discovery rate (FDR) was set to 0.01 for protein- and peptide-level identifications. 2 missed cleavage were allowed, and the minimum peptide length was set to 8 amino acids. Variable modifications: lysine monomethylation (+ 14.016), dimethylation (+ 28.031 Da), trimethylation (+ 42.046 Da) and acetylation (+ 42.010 Da), D3-acetylation/propionylation (+ 45.0294/+ 56.0262 Da), lysine monomethylation with D3-acetylation/propionylation (+ 59.0454/+ 70.0422), dimethylation, trimethylation and lysine acetylation.

**Western Blot** Nanoparticle treated cells were lysed in Laemmli buffer. Cell lysates were incubated at 97 ℃ for 2 min followed by 30 seconds sonication and another 5 min incubation at 97 ℃. The lysates were then centrifuged at 20,817 g for 10 min to remove nanoparticles and insolubilities. 10 μg lysate was separated on 15% SDS-PAGE gel and transferred to PVDF membrane with pore size of 0.2 μm. The membranes were then blocked with 5% skim milk in TBS-T buffer (150 mM NaCl, 10 mM Trizma base, 0.1% Tween-20, pH 7.5) for 1 hour. The membranes were further incubated with primary antibody diluted in blocking buffer at 4 ℃ overnight. After 4 times washes with TBS-T buffer for 10 min, the membranes were incubated with HRP-labelled secondary antibody for 1 hour and soaked four times in TBS-T buffer for 10 min. The membranes were developed with PierceTM ECL Western blotting substrate mix solution and visualised in Syngene G:BOX imaging system.

**ChIP and ChIP-seq** ChIP experiment was done following the protocol described in literature (4). Briefly, a total of 1×10^8^ A549 cells were 1% formaldehyde-fixed, lysed and sonicated for 16 cycles (30 s sonication 55 s rest) in a Bioruptor sonicator (Diagenode, Denville, NJ, USA). Supernatants were precleared with pierce^TM^ protein A/G magnetic beads (Thermo Scientific). Chromatin fragments were immunoprecipitated by using rabbit H3K27me3 antibody or normal rabbit IgG antibody (Cell Signalling) and purified using QIAquick PCR purification columns (Qiagen, Hilden, Germany). DNA samples were shipped to BGI on dry ice for ChIP-seq library preparation and sequencing. Samples’ quality was checked by Bioanalyzer (Agilent 2100) before sequencing, sequencing was performed using DNBseq system (BGI) with each library sequenced in a 50 base pairs single-read run with 50 million reads for each sample. Reads were aligned to the Homo sapiens genome (hg 38) using Bowtie 2 (5) with default parameters. Peak calling was performed by using MACS 3 (6) with command for broad peaks. ChIP-seq was performed in two biological replicates and Diffbind (7) was used to find the consensus peaks and the differential binding sites for each treatment. The differential binding sites annotated genes were used for further network and ontogeny analysis by ChIPseeker (8), clusterProfiler (9) in R package and online website metascape (10).

**SI References**

1. Talamini, L.; Violatto, M. B.; Cai, Q.; Monopoli, M. P.; Kantner, K.; Krpetic, Z.; Perez-Potti, A.; Cookman, J.; Garry, D.; C, P. S.; Boselli, L.; Pelaz, B.; Serchi, T.; Cambier, S.; Gutleb, A. C.; Feliu, N.; Yan, Y.; Salmona, M.; Parak, W. J.; Dawson, K. A.; Bigini, P., Influence of Size and Shape on the Anatomical Distribution of Endotoxin-Free Gold Nanoparticles. *ACS Nano* 2017*, 11 (6)*, 5519-5529.
2. Boselli, L.; Lopez, H.; Zhang, W.; Cai, Q.; Giannone, V. A.; Li, J.; Moura, A.; de Araujo, J. M.; Cookman, J.; Castagnola, V.; Yan, Y.; Dawson, K. A., Classification and biological identity of complex nano shapes. *Communications Materials* **2020,** *1 (1).*
3. Feller, C.; Forné, I.; Imhof, A.; Becker, P. B., Global and specific responses of the histone acetylome to systematic perturbation. *Molecular cell* **2015,** *57 (3),* 559-571.
4. Mukhopadhyay, A.; Deplancke, B.; Walhout, A. J. M.; Tissenbaum, H. A., Chromatin immunoprecipitation (ChIP) coupled to detection by quantitative real-time PCR to study transcription factor binding to DNA in Caenorhabditis elegans. *Nature protocols* **2008,** *3 (4),* 698-709.
5. Langmead, B.; Salzberg, S. L., Fast gapped-read alignment with Bowtie 2. *Nature Methods* **2012,** *9 (4),* 357-359.
6. Zhang, Y.; Liu, T.; Meyer, C. A.; Eeckhoute, J.; Johnson, D. S.; Bernstein, B. E.; Nusbaum, C.; Myers, R. M.; Brown, M.; Li, W.; Liu, X. S., Model-based Analysis of ChIP-Seq (MACS). *Genome Biology* **2008,** *9 (9),* R137.
7. Ross-Innes, C. S.; Stark, R.; Teschendorff, A. E.; Holmes, K. A.; Ali, H. R.; Dunning, M. J.; Brown, G. D.; Gojis, O.; Ellis, I. O.; Green, A. R.; Ali, S.; Chin, S.-F.; Palmieri, C.; Caldas, C.; Carroll, J. S., Differential oestrogen receptor binding is associated with clinical outcome in breast cancer. *Nature* **2012,** *481 (7381),* 389-393.
8. Yu, G.; Wang, L.-G.; He, Q.-Y., ChIPseeker: an R/Bioconductor package for ChIP peak annotation, comparison and visualization. *Bioinformatics* **2015,** *31 (14),* 2382-2383.
9. Yu, G.; Wang, L.-G.; Han, Y.; He, Q.-Y., clusterProfiler: an R package for comparing biological themes among gene clusters. *OMICS* **2012,** *16 (5),* 284-287.
10. Zhou, Y.; Zhou, B.; Pache, L.; Chang, M.; Khodabakhshi, A. H.; Tanaseichuk, O.; Benner, C.; Chanda, S. K., Metascape provides a biologist-oriented resource for the analysis of systems-level datasets. *Nature communications* **2019,** *10 (1),* 1523-1523.
